# Infectious disease markers in blood donors at Central Referral Hospital, Gangtok, Sikkim

**DOI:** 10.4103/0973-6247.59392

**Published:** 2010-01

**Authors:** Luna Adhikari, Dharmraj Bhatta, Dechen C. Tsering, Dhruva Kr Sharma, Ranabir Pal, Amlan Gupta

**Affiliations:** Associate; 1 Department of Microbiology, Sikkim-Manipal Institute of Medical Sciences (SMIMS) and Central Referral Hospital (CRH), 5^th^ Mile Tadong, Gangtok, Sikkim - 737 102, India; 2 Department of Medical Microbiology, Sikkim-Manipal Institute of Medical Sciences (SMIMS) and Central Referral Hospital (CRH), 5^th^ Mile Tadong, Gangtok, Sikkim - 737 102, India; 3 Sikkim-Manipal Institute of Medical Sciences (SMIMS) and Central Referral Hospital (CRH), 5^th^ Mile Tadong, Gangtok, Sikkim - 737 102, India; 4 Department of Pathology, Sikkim-Manipal Institute of Medical Sciences (SMIMS) and Central Referral Hospital (CRH), 5^th^ Mile Tadong, Gangtok, Sikkim - 737 102, India; 5 Department of Community Medicine, Sikkim-Manipal Institute of Medical Sciences (SMIMS) and Central Referral Hospital (CRH), 5^th^ Mile Tadong, Gangtok, Sikkim - 737 102, India

Sir,

A safe blood is a critical component in improving health care and in preventing the spread of infectious diseases globally. Yet the quality and safety of blood transfusion is still a concern for a health-care personnel.

The extent of infectious disease markers of major blood-borne pathogens [hepatitis B (HBV), hepatitis C (HCV), human immunodeficiency virus (HIV), syphilis and malarial parasite] was observed as with age and sex distribution for the donor population of the blood bank of a tertiary care hospital of Gangtok during 2001-2008.

Of the 3735 donors, 2423 (64.87%) were replacement and 1312 (35.13%) voluntary donors; male donors were 3434 (91.94%) and females were 301 (8.06%) with overall seroprevalence of major blood-borne pathogens (HIV, HBV, HCV and Syphilis) was 1.63%. The sero-positivity of HIV, HBsAg, anti- HCV, and syphilis was 0.32, 0.78, 0.27 and 0.27%, respectively. Unlike other studies,[1,2] which showed a lower prevalence of HIV, HBsAg, and anti-HCV in voluntary donors, we observed a higher HIV, HBsAg, and anti-HCV sero- positivity in voluntary donors as compared to replacement donors, though the difference in prevalence in these two groups was not statistically significant [Chi-square test (with Yates correction) = 0.02974 (*P* = 0.10); 0.01495; (*P* = 0.9027); 1.738 (*P* = 0.1874) respectively]. Whereas syphilis positivity was found to be higher in replacement donors as compared to voluntary donors though not statistically significant [Chi-square test (with Yates correction) = 0.4513 (*P* = 0.5017)], which was in agreement with other studies.[1,2] HIV and anti-HCV sero-positivity was nil from 2001 to 2005 among our donors. There was no significant change in the prevalence of HIV, HBsAg, HCV, and syphilis positivity over the period (Chi - square for trend = 5.913, *P* = 0.0120; 0.01728, *P* = 0.6776; 2.440, *P* = 0.1183; 0.04003, *P* = 0.8414, respectively).

There was no HIV, HCV and syphilis positive case among female donors in our study population. The HBV positivity was the only infection found in one female donor in our total female donors in the age group of 21-40, which was statistically significant (*P* = 0.0062). There were no HIV and syphilis positive cases below the age of 21 years. No sample showed malarial parasites. There was not a single case positive for co-infection with two or more infectious agents.

Though the screening tests used for these infections were not 100% sensitive or specific,[3,4] and the approximation of the infectious disease markers in our donor population was less, there is no scope of complacency.
